# *No Pass Laws Here!* Internal Border Controls and the Global ‘Hostile Environment’

**DOI:** 10.1177/00380385221122518

**Published:** 2022-10-25

**Authors:** Kathryn Medien

**Affiliations:** The Open University, UK

**Keywords:** anti-imperialism, anti-racism, borders, internal border controls, migration, racial capitalism, state racism

## Abstract

This article explores internal border controls in 1980s Britain, examining how they were conceptualised and resisted by a group of activists, the *No Pass Laws Here!* Group. Drawing on archival research conducted at the Hull History Centre and the Institute of Race Relations and focusing analysis on the Group’s public-facing information leaflets and bulletins, this article explores how internal border controls created differentiated access to employment and the welfare state, targeting migrant and racialised residents and citizens. The *No Pass Laws Here!* Group’s framing and analysis, in particular their use of pass laws as a frame through which to apprehend the spread of internal border controls, this article argues, allows us to draw out the continuities between policies developed to maintain colonial rule and those present in the metropole.

## Introduction

Internal border controls – that is, citizenship and residency checks designed to sort, exclude and govern within a nation state’s borders, rather than at ports of entry – in Britain today are shaped by what is termed the ‘hostile environment’, more recently renamed as the ‘compliant environment’. The term ‘hostile environment’ was introduced by then Home Secretary Theresa May in 2012, and its associated policies were developed by the ‘Hostile Environment Working Group’ and introduced through the 2014 and 2016 Immigration Acts. Together, these acts have placed internal border controls within a wide array of public and private services and welfare institutions; housing, healthcare, financial services, education, driving licensing, legal aid, domestic violence services and elsewhere (see Corporate Watch, 2018; Jones et al., 2017; Social Scientists Against the Hostile Environment, 2020). Specifically designed to target migrants who live precariously without recourse to public funds, and to restrict undocumented migrants’ access to public and private services and employment, these policies are sustained through passport checks that turn workers into border guards, data sharing between various government departments and the Home Office, and broader racial profiling and questioning. For example, the Immigration Act 2014 provided power to revoke UK driving licences held by undocumented migrants and, by 2016, led to over 16,000 licences being revoked. The Immigration Act 2016 built upon these powers, providing immigration officers and the police force with the power to search people and premises in order to seize driving licences held by undocumented migrants. The 2016 Act also led to the creation of a new criminal offence, ‘driving whilst unlawfully present in the UK’ (Home Office, 2016), which carries a custodial sentence of up to six months. Thus, through data sharing and collaboration between government departments and increased policing and surveillance, the hostile environment policies install internal border controls within everyday spheres of public life.

The introduction of these internal border policies has had far-reaching effects on the lives of racialised migrants in Britain, including long-term Black residents who form part of the Windrush generation, and have been met with ongoing resistance.^1^ Following their introduction, a range of sociological, migration studies and socio-legal scholarship has sought to examine the contours of these policies, their effects on the lives of those targeted and the various ways that they are being resisted (Coddington, 2021; Cole, 2019; El-Enany, 2020; Griffiths and Yeo, 2021; Redclift and Rajina, 2021; Shahvisi, 2019). Through this literature, we can gain a better understanding of how internal borders today operate to expand the contours of ‘illegality’ (Tyler, 2018), and thus ‘undermine a naturalised sense of entitlement to citizenship rights to a growing section of the population’ (Yuval-Davis et al., 2018: 239–240). Here scholars have underscored the centrality of racism and racialisation to these practices of ‘everyday bordering’ (Yuval-Davis et al., 2018) and have traced and connected today’s hostile environment policies to their earlier British counterparts, including the Aliens Act 1905, the Commonwealth Immigrants Act 1962 and the Commonwealth Immigration Act 1968, among others. As Erel et al. (2016: 1343) have argued, the exclusionary and othering logics that underpin border controls are historically shaped through ‘race as a political project rooted in colonialism and imperialism’ (see also Bhui, 2016). Thus, internal border controls are practices of racist classification and exclusion, even as their political focus can expand and shift to encompass populations (often ambivalently) categorised as ‘white’ witnessed, for example, in the run up to and aftermath of the 2016 Brexit referendum. In this sense, following De Noronha (2019: 2419), ‘border regimes are central to the production, or reconfiguration, of race as a social relation and system of difference’.

This article seeks to contribute to this literature by examining an earlier 1980s history of racialised internal border controls, and by documenting how these earlier internal borders were understood and resisted by migrant and anti-racist community organisations. Drawing on archival research and focusing on the work of one particular group, the *No Pass Laws Here!* Group (NPLH!), this article documents the incursion of border controls into everyday life in early 1980s Britain. Through an analysis of the *No Pass Laws Here!* Group’s bulletins, information booklets and public correspondence, I suggest that their archive offers a series of empirical case studies that demonstrate the older and far-reaching effects of racist internal border controls within Britain. Furthermore, in drawing on the group’s framing and analysis, I propose that they produced an analytic of internal passport controls that may allow us to better understand how internal borders function as a technology of state racism that divides the working classes through racialisation, thus perpetuating a system of racialised capitalism that facilitates the (re)production of exclusionary nationalisms. Linking internal border controls here to regimes of racialised surveillance and control elsewhere, most notably the pass law system of Apartheid South Africa, the group offer a way of analysing internal border controls that does not rely on ‘methodological nationalism’ (Wilder, 2015: 3–4), but instead helps us conceptualise internal border controls as both a reconfiguration of empire within Britain and in direct relation to colonial and racist subjugation elsewhere. The result of such an analytic, I suggest, is an understanding of internal border controls as functioning to secure the reproduction of racialised capitalism, and an attendant framework for resistance that is distinctly working class, anti-racist and anti-imperialist in its orientation.

This article proceeds by outlining the historical context within which *No Pass Laws Here!* emerged and offers an overview of the scope of their activities. Second, I examine the *No Pass Laws Here!* Group’s bulletins in more detail, drawing out the ways that their contents help us better understand how internal borders centrally support the racialised organisation and division of labour. Third, I turn to the framing of the group, highlighting how the language of pass laws enabled the group to situate their anti-racist analysis in relation to other anti-racist and anti-colonial movements in Britain and elsewhere. This framing, I suggest, allows us to draw out the continuities and flows between policies developed under colonial rule and those present in the metropole, as well as the persistence of colonial modes of governance today. In so doing, I consider how ‘Pass Laws’ as an analytic might enhance our understanding of internal border control as a technology of racialised governance globally. To conclude, I reflect upon what current struggles against internal borders may learn from this history of anti-border resistance.

## Methodology

This article draws on archival research conducted at the Hull History Centre (Hull) and the Institute of Race Relations (London) in 2019 and 2020. While visiting these archives, I reviewed specific collections that held materials related to the *No Pass Laws Here!* campaign, which included meeting minutes and other notes, private correspondence between the *No Pass Laws Here!* Group and other organisations and individuals, and public-facing leaflets, information booklets and bulletins produced by the group. In the article, I focus my analysis on the contents of public-facing information booklets, leaflets and bulletins produced by the *No Pass Laws Here!* Group. This includes nine NPLH! bulletins circulated between 1982 and 1987, four information leaflets targeted at different status categories (e.g. overseas students, EEC nationals) containing advice for accessing state services, a conference report and three two-sided leaflets produced by *No Pass Laws Here!* and *No Pass Laws in the North West!*. After carefully reading all public-facing materials, I narrowed my focus to items that specifically detailed policies and instances of resistance to internal border controls, rather than, for example, book reviews or notices publicising meetings or events. Here I looked at common themes, the political language used and noted particular cases and policies of significance. In so doing, I was able to gain an understanding of the breadth of internal border controls in 1980s Britain, their impact on migrant and racialised communities and how these border controls were conceptualised and critiqued by *No Pass Laws Here!* as a collective.

Importantly, archives are not neutral sites awaiting discovery, rather as Moore et al. (2016: 24) note, they are ‘marked by selections, occlusions, exclusions, partiality, fragmentation, and these pressures impact on what remains, how it is organised, accessed and worked on’. Furthermore, archival research is shaped by the researcher’s ‘own sense of what is important, interesting, and how it should be pursued’ (Moore et al., 2016: 24). In this article, I have chosen to focus my analysis on the *No Pass Laws Here!* Group’s public-facing materials, rather than private correspondence, internal meeting minutes or draft articles and analyses held in the archives. This decision was made because the aim of this article is to outline and analyse the agreed upon political framing used by the group and explore how they documented and circulated information regarding internal border controls in 1980s Britain. In so doing, I ask how these public-facing materials may offer us fruitful lines of inquiry in the present. This is best achieved, I would suggest, through a focus on outward-facing documents that present collectively agreed upon framings and documentation. In making these partial selections, my aim is to open the *No Pass Laws Here!* archives for discussion and analysis, asking how they may help us grapple with internal border controls in the present.

In some of the materials produced by the *No Pass Laws Here!* Group and presented in this article, ‘Black’ is used within a framework of ‘political Blackness’ and used to denote racialised or non-white populations in general, rather than Black people specifically. However, throughout the *No Pass Laws Here!* materials, the ethnic and national groups specifically targeted by internal border controls are also named (e.g. Ghanaian, Hong Kongers, Bengali). The use of the term Black within a framework of political Blackness is a specific and contested anti-imperialist and multi-racial political framework that was used among certain anti-racist organisations within Britain during the 1960s, 1970s and 1980s (Alexander, 2018; Bourne, 2016; Brah, 1996; Narayan, 2019). Indeed, writing within the context of the 1960s and 1970s British Black Power (BBP) movement, Narayan (2019: 953) notes that the ‘“Black” signifier of political blackness functioned to plug BBP activism into a global circuit of anti-imperialist activity in the Third World’ (2019: 953), and thus served to ‘highlight the connections of an exploitative state in the UK and an exploitative global capitalist system abroad’ (2019: 959). However, the framework and language of political Blackness has been critiqued for erasing the specificities of anti-Black racism and obscuring various differences between ethnic and religious communities (Abbas, 2020; Modood, 1994). In light of this, throughout this article I seek to both draw out the anti-imperialist praxis that was central to the framing of the *No Pass Laws Here!* campaign, while also naming the specific communities who were targeted by internal border controls within 1980s Britain.

## No Pass Laws Here!

According to meeting minutes and private correspondence contained within the Hull History Centre archive, the *No Pass Laws Here!* Group formed in 1981 out of a collaboration between already existing organisations and individuals. Based at the Joint Council for the Welfare of Immigrants (JCWI) offices on Theobalds Road in London, the group initially drew its members from the experienced campaigners and case workers of the JCWI and its associated groups. Indeed, in the early 1982 bulletins, the group stated that, ‘In association with the Joint Council for the Welfare of Immigrants the *No Pass Laws Here!* Group has been collecting instances of the increasing amount of surveillance, control and harassment of immigrant and black residents in Britain.’^2^ However, in their later bulletins from 1983 onwards, the *No Pass Laws Here!* Group had expanded, listing their constituent members as including the following: the Joint Council for the Welfare of Immigrants, Hackney Anti-Deportation Campaign, Migrants Action Group, Islington Law Centre, Greenwich Welfare Rights, Lambeth Welfare Rights, African Refugee Housing Action Group, Hillingdon Legal Resource Centre, Child Poverty Action Group, among others.^3^

Together, this coalition of groups, organising under the campaign *No Pass Laws Here!*, sought to ‘monitor and report on the operation of internal controls’ and campaigned for the following demands:

- An end to passport and immigration checks
- An end to immigration raids
- No denial or restriction of services/benefits/jobs because of immigration status
- No passing of confidential information between central government departments and central government departments and other institutions
- No collaboration with racist passport or immigration status checks.^4^

The group defined their remit as seeking to raise awareness of the spread of internal border controls in Britain and they sought to do this through issuing ‘bulletins at regular intervals, cataloguing examples of both infringements and opposition to them’^5^ and offered information to those navigating internal border controls through the production of advice pamphlets on the implications of internal border controls for specific benefits or categories of immigration status. From the correspondence contained within the archives, it is clear that the bulletins and pamphlets had wide reach. Indeed, letters from individuals and groups requesting copies of *No Pass Laws Here!* bulletins came from across the UK, including from Sheffield, Cambridge, Belfast, Birmingham, London and Mid Glamorgan. Each bulletin, a four-page double-sided A4 document, was structured around an editorial analysis of a specific policy or event, one or two shorter articles that detailed, for example, policy updates or reports on immigration raids, a call-out for information and a final section titled ‘Area Roundup’ that provided short overviews of local cases, updates from regional campaign groups and information on legal and policy changes. The March 1982 bulletin, for example, contained a long-form article titled ‘Racism in social security’, which detailed how changes to the Social Security Act 1980 had paved the way for both the refusal of benefit to immigrant claimants and now required ‘“foreign” looking or sounding applicants’^6^ to produce their passports in order to establish benefit eligibility. The same bulletin also contained an article detailing a passport raid in Mitcham, and the ‘Area Roundup’ covered internal borders within social housing in Birmingham, passport checks in schools in Nottingham and an update from the Black Women’s Health Campaign, who were seeking to ‘monitor racist policies and practices in clinics and hospitals in Tower Hamlets, from forced sterilization and Depo-Provera injections to passport controls’.^7^

In documenting and analysing the policy and legal changes that were making access to daily life in 1980s Britain conditional on immigration and nationality status and attendant racial profiling, the bulletins produced by the *No Pass Laws Here!* Group offer us a rich insight into the existence and function of internal border controls in 1980s Britain. These borders included, according to a leaflet produced by the Group, the following:

- passport ‘raids’ at their workplaces
- demands for passports when applying for jobs, local authority housing or NHS treatment
- the risk of being reported to the Home Office by registrars of marriage and hospital administrators
- checks on their immigration status when applying for national insurance cards or supplementary benefits
- visits to their home by police officers to check whether they have ‘suitable accommodation’ for dependents
- confidential reports by the police to the Home Office on their immigration status if they are convicted of offences
- questionnaires about the validity of their marriage if they apply for maternity grants
- reports to the Home Office by college authorities on their academic progress if they are students.^8^

This increasing array of internal border controls, the *No Pass Laws Here!* Group argued, could be situated within a number of shifts in immigration and nationality policy. These included the 1971 Immigration Act, which, the group noted in their May 1987 bulletin, had ‘shifted the emphasis of immigration control away from UK ports of entry and overseas posts over to institutions and agencies inside this country’.^9^ The more recent British Nationality Act 1981 built upon the 1971 Act. The 1981 Act reclassified the category ‘Citizenship of the United Kingdom and Colonies’ into three new categories, resulting in the removal of formerly colonised populations from the category of British citizenship and the defining of citizenship through lines of descent to the post-colonial territory ‘Britain’. As a result, Tyler (2010: 62) has argued that the 1981 Act was ‘designed to define, limit and remove the entitlements to citizenship from British nationals in the Commonwealth (the former colonies) thereby restricting immigration to the British Isles and creating “aliens” within the borders of the nation state’. In order to identify and police these newly created categories of ‘alien’ within Britain’s post-colonial borders, and often entwined with discourses of austerity and resource scarcity, a number of internal border controls arose. These included the 1982 introduction of passport checks and charges for migrant NHS care and the 1985 Housing Act, which stated that housing provision would be unavailable to those ‘without recourse to public funds’. In the section that follows, I turn to examine the content of the *No Pass Laws Here!* Group’s public-facing materials in more detail, specifically considering how they understood and analysed the function of internal border controls and the centrality of class analysis to their politics.

## Internal Border Controls and the Racial Organisation of Workers

The public-facing materials produced by the *No Pass Laws Here!* campaign offer us a detailed documentation and analysis of extensive internal border controls in Britain. In this section, I examine the content of these materials in more detail and consider how the group’s documentation allows us to better understand how internal border controls function – that is, how they work to demarcate and survey groups, transform social relations and create or reinforce hierarchies and exclusions. In so doing, I suggest that the *No Pass Laws Here!* Group’s analysis articulates internal border controls as working to divide the working class through racialisation. Racialisation, as process or technology that can function to divide workers, has been examined in great detail by numerous scholars (Bhattacharyya, 2018; Du Bois, 1998, 2008; Itzigsohn and Brown, 2020; Lowe, 2015; Mondon and Winter, 2019; Narayan, 2017, 2019; Robinson, 2020; Virdee, 2014). In his foundational work on imperialism and the racialised nature of capitalism, Du Bois (2008) demonstrated how white working classes in southern US states came to accept their class position precisely because it was embedded in a racialised hierarchy that placed them above, and at times in direct supervision of, Black workers (see also Du Bois, 1998: 12). Rather than a specific analysis of the USA, the racialised nature of class was always international and, ‘for Du Bois, always intersects with colonialism and the global color line . . . race fractures class interests and hinders collective action’ (Itzigsohn and Brown, 2020: 67). In this section, I build upon this literature, exploring how, as the *No Pass Laws Here!* Group articulated it in their April 1982 bulletin, ‘internal immigration controls [are] designed to intimidate and frighten black and migrant workers [and] make it more difficult to develop united working-class organisation’.^10^ In so doing, I hope to demonstrate that internal border controls function as technologies that actively perpetuate and (re)produce race as a system of social fragmentation, division and difference.

Internal border controls within the welfare state were central to the internal border regime in 1980s Britain, functioning as both a key mechanism through which new immigration and nationality legislation could be policed and as a means to implement so-called cost saving measures that, in effect, specifically targeted racialised and/or migrant residents and citizens. These internal borders sought to mark out racialised subjects for questioning, delegitimising their access to state provisions and services such that ‘a would-be claimant who looks or sounds “foreign” is the object of suspicion and is subjected to in-depth questioning and investigations from the DHSS through to the Home Office and perhaps Inland Revenue’.^11^ One effect of this questioning, the *No Pass Laws Here!* campaign argued, was ‘to discourage them from applying for supplementary benefit for fear that the bottomless pit of questions about illegal entry and deception be opened’.^12^ More broadly, the group argued that the incursion of border controls within the British welfare state perpetuated a myth that migrant and racialised communities were over-reliant on the state, participating in benefit tourism and committing benefit fraud. This myth was perpetuated despite, as the May 1987 *No Pass Laws Here!* bulletin argued, ‘the evidence from voluntary organisations, local councils and academic studies [that] shows that Black people are far less likely to claim to make claims for benefits. And where they do make claims, they claim to little.’^13^ This process of racialised differentiation, whereby potential welfare recipients are sorted through racial profiling and passport checks, was policed by public sector workers turned border guards. In so doing, such internal border controls functioned to create a racial hierarchy within the working class and perpetuated notions that white residents were more entitled to and deserving of benefits and state services than their racialised counterparts.

In their first bulletin, published in January 1982, the group examined the proposed introduction of NHS charges for overseas visitors. The proposed policy, which later came into effect in October 1982, sought to introduce passport checks and charges for overseas visitors’ NHS treatment, with responsibility placed on healthcare providers to recuperate outstanding debt. This was to be done through an amendment to section 121 of the NHS Act 1977 and was justified through claims of alleged widespread abuse of the health service by foreign nationals. As a result, the government suggested that the policy would save the NHS £5 million a year. Detailing the proposal in their bulletin, the *No Pass Laws Here!* Group made a number of observations and raised key concerns with the proposed legislation, namely that:

the scheme could also turn NHS staff into agents of the Immigration Department of the Home Office. There are already cases where hospitals have to sort information from the Home Office before providing treatment, and the new scheme would require such contact in some cases. There would therefore be a link between health service entitlement and a close scrutiny of immigration status, a link which would inhibit anyone unsure of his or her immigration status from seeking treatment. This would not only be detrimental to the health of that person, but possibly also to the health of others. Black people will, inevitably, be the first to suffer from this scheme.^14^

The *No Pass Laws Here!* Group argued that the proposed changes to NHS care would ‘inevitably be racially discriminatory’ and would function as ‘yet another area of surveillance on their [racialised residents’ and citizens’] everyday lives’.^15^ Importantly, the group’s analysis of the proposed passport checks and charges for migrant healthcare chimes with more recent critiques of current NHS charging and data sharing regulations under the hostile environment. Here campaigners have argued that the introduction of immigration checks and charges for NHS treatment target racialised and migrant communities and gain their popular justification through discourses of health tourism, a ‘baseless myth to support the supposed need to exclude people from the NHS’ (Medact, 2020: 8; see also Medien, 2021).

While immigration controls within the welfare state were barring racialised residents and citizens from accessing welfare support, formal and informal controls within the workforce were also making it increasingly difficult to enter the workforce. The *No Pass Laws Here!* Group noted that the ‘myth that unemployment is caused by immigration is now well entrenched in Britain’s official policy’, which masked the fact that ‘since 1975 every applicant for a National Insurance number must produce proof of identity’, that ‘Job Centres often seek evidence that there are no restrictions on taking employment before they will register someone as unemployed’ and that ‘National Insurance records are widely used by the police and the Immigration Service to detect unauthorised workers and illegal immigrants’.^16^ In certain instances, the *No Pass Laws Here!* Group noted that internal border control within the workplace sought to distinguish white workers from those who were racialised and/or migrant, which in turn had an impact on hiring practices and union activities. As the October 1982 bulletin notes:

as a consequence of immigration raids at workplaces, more employers have asked workers of foreign descent to produce passports to prove that they are here legally and have permission to work . . . [in] some cases there have been attempts to ensure the cooperation also of shop stewards and to divide the work forces on lines of colour.^17^

Through the state perpetuation of immigration ‘myths’ and government ‘attempts to turn British workers in state agencies into watchdogs over the rest of the working class’,^18^*No Pass Laws Here!* understood internal borders as an extensive state infrastructure that differentiated access to the welfare state and employment, while also fracturing working-class relations through turning public sector workers into border guards and perpetuating racialising notions that non-white residents were ‘gate crashers’, ‘fraudsters’ and undeserving of the state support. Through the cases documented by the campaign and framed through the language of pass laws – a framing that, as I argue in the next section, always gestures towards a relationality and solidarity with subjugation elsewhere – internal border controls can be understood as functioning to fracture society through racialisation, while restricting migrant communities’ access to vital resources. Such analysis contributes to our understanding of anti-racist history and analysis in the UK, offering insights into how activists connected internal border controls to class relations and state racism.

## *No Pass Laws Here!* as Analytic

In their public-facing materials, the *No Pass Laws Here!* Group offered a situated political analysis of British state racism, focusing their specific attention on the effects of and resistance to internal border controls. However, their framing also situated internal borders in Britain in relation to racialised control and surveillance elsewhere. The group took their name – *No Pass Laws Here!* – from the pass law system of colonial Apartheid South Africa.^19^ In so doing, the group drew lines of relation, but not conflation, between racist supremacist movement control in Britain and that taking place under South African Apartheid rule. In this section, I move from an analysis of internal border controls in 1980s Britain to consider the group’s framing at the scale of the international. In so doing, I consider how the framing of ‘pass laws’ allows us to apprehend internal border controls as a racialising and colonial technology that, while manifesting differently in differing locations, signals the endurance of colonial modes of governance in differing locales.

South Africa’s pass law system dates back to the Cape Colony where pass documents were used to control and restrict the movements of Black South Africans. The technology of pass laws developed, expanded and persisted until their repeal in 1986. Under Apartheid, pass laws required Black South African workers, initially men, to carry internal passports with them at all times in order to access employment, services and land. Operating as a regime of labour and mobility control that sought to racially manage urbanisation, while also creating an internal infrastructure for deportation and incarceration, pass laws ‘attempted to manage the “threat” posed by black people by incarcerating them in zones of containment while also enabling the control and policed exploitation of black people as workers, on which the country was dependent’ (Besteman, 2019: 28; see also Lipton, 1986; Wolpe, 1990). While ‘white power and geographic segregation remain underpinned by the pass laws’ (Savage, 1986: 183), they also functioned to (re)produce geographies of heteronormative and gendered divisions of labour. Internal passports were primarily issued to men until 1956, while the regime spatially confined Black women’s mobility and working lives to rural areas, informal sectors and unwaged labour, meaning that ‘pass laws were one mechanism for the social construction of gender’ (Barnes, 1997: 61). As such, the pass law system operated as a regulatory internal security apparatus that sought to maintain racial segregation and dispossession within a white supremacist state, while also securing the provision of cheap and precarious Black labour – in short, pass laws functioned to maintain and perpetuate a system of patriarchal racialised and colonial capitalism.

South Africa’s apartheid pass law system, as a colonial technology and socio-legal apparatus, laid the foundation for, and has been positioned in relation to, a number of contemporary regimes of racialised labour and movement control globally. Similar to South Africa, the state of Israel’s permit regime has its foundations in British Mandate colonial policy (Berda, 2017), and parallels have been drawn ‘between the South African “pass laws” and the permit regime that the State of Israel uses to classify, track, and control the movement of Palestinians from the occupied territories’ (Clarno, 2017: 4). Hahamovitch (2013: 18) has detailed how South Africa’s pass law regime provided a blueprint for a variety of ‘guest worker’ programmes across the Global North, where states in North America and Europe adopted temporary visa programmes in order to manage ‘the desire to admit immigrant workers and the urge to expel them’. In this sense, we might understand South Africa’s pass law regime and its afterlives, while translating and manifesting differently in differing contexts, as providing a set of logics or technologies for governance that have allowed for the intricate racialised governance of migration, internal movement control and labour exploitation.

In their early public-facing materials, the *No Pass Laws Here!* Group were clear that the increasing array of internal border controls in Britain, while operating as a racist system of welfare restriction, movement control and surveillance, were not identical or even comparable to South Africa’s apartheid pass law regime. As they wrote in a two-sided leaflet:

NO PASS LAWS HERE!

That’s a statement of fact. Britain has no pass laws similar to those of South Africa.

But Britain does subject its black residents to internal surveillance, control and harassment of many kinds.^20^

Thus, their name and demand, *No Pass Laws Here!*, differentiated Britain’s internal borders from South Africa’s pass law regime at the same time as, I would argue, they signalled continuities between these modes of governance. Recognising that the increasing array of internal borders – within the NHS, social security, housing, employment and elsewhere – required racialised residents to carry their passports as a means to access quotidian life, the demand – *No Pass Laws Here!* – signalled their concern that a ‘colour bar’ was being formalised through immigration legislation and welfare policy resulting in differentiated access to resources. Indeed, in a May 1987 article that debunked and critiqued claims being made by the Home Office and tabloid press that Ghanaian and Nigerian migrants were engaged in widespread benefit fraud, *No Pass Laws Here!* noted that:

The effect of this informal system of internal immigration control is to place whole sections of the Black community under surveillance. On top of this the ‘no recourse to public funds’ criteria in the immigration rules treats them as ‘gate crashers’ on the welfare state. In turn this has led to a collection of largely unlawful or dubious practices which deny access to benefits and services. But ultimately internal immigration control is a threat to the rights of the Black people to live in this country at all. Because everyone who is Black, whether they are subject to immigration control or not, know that their right to be here is questioned whenever they are asked about their place of birth, nationality, immigration status or to produce a passport as proof of any of these . . . In short, it is a system of ‘pass laws’.^21^

As the above passage attests, the *No Pass Laws Here!* Group recognised the central role that internal border controls played facilitating a precarious and conditional system of residency and citizenship for migrant and/or racialised communities, underscoring the British state’s ability to undermine and deprive those communities of their very right to reside in Britain. Furthermore, such analysis also alludes to the relationship between racialisation, nationalism, welfare and capitalism, whereby the British state admits a replaceable and precarious migrant workforce for labour exploitation, at the same time as it restricts national welfare and state services from such populations. Here we can see the lines of relation or continuities between Apartheid-era pass laws and contemporary internal border controls in Britain, which develops this article’s argument that the *No Pass Laws Here!* Group help us conceptualise internal border controls as a reconfiguration or coming home of empire within Britain.

Importantly, the use of pass laws as an equivalence to apprehend Britain’s internal border controls was not one-way nor was it unique to this campaign. In January 1981, for example, the UK-based Azania Solidarity Campaign wrote to the *No Pass Laws Here!* campaign inviting them to speak at a memorial event being held to commemorate the Sharpsville Massacre of 1960, which had taken place at an anti-pass law demonstration, alongside speakers from the ANC (African National Congress), PAC (Pan-African Congress) and BCMA (Black Consciousness Movement of Azania). The letter reads, ‘We sincerely hope that you will accept our invitation. It would enable the link to be made between the anti-pass laws demonstration there with contemporary developments in Britain.’^22^ Furthermore, the language of pass laws was taken up by other campaigns and activists. In his 1981 essay ‘From resistance to rebellion: Asian and Afro-Caribbean struggles in Britain’, A Sivanandan (1981) argued that the 1981 Nationality Act was formalising an infrastructure of internal passport controls that would make housing, welfare, and employment contingent on passport checks. In so doing, he argued that ‘Britain was effectively moving to a pass-law society’ (1981: 147).^23^

The circulation of the language of ‘pass laws’ among the anti-racist left in 1980s Britain allowed activists and intellectuals to scale their analyses of internal surveillance and movement control from the national to the global. Here ‘pass laws’ provided a frame through which to understand technologies of governance associated with colonial and apartheid rule in relation to the increasingly restrictive immigration and nationality policies in post-colonial Britain. This framing built upon earlier traditions of anti-racist organising that took a distinctly anti-imperial stance (Ashe et al., 2016; Bebber, 2015; Bryan et al., 2018; Davies, 2008; Narayan, 2019; Sivanandan, 1981), and allowed the groups to position anti-racist resistance in Britain as part of a broader global struggle against state racism and imperialism. Furthermore, I would suggest that the use of this framing allows us to draw out the material continuities between restrictive technologies of governance globally and underscores the vital relationship that migration and border regimes play in the perpetuation of regimes of racialised capitalism. In this sense, rather than a relic of a past apartheid system, we can understand pass laws as a racist and segregationist technology that also characterise and shape the colonial metropole and whose logics persist today. Within this context, I want to suggest that *No Pass Laws Here!* as a demand provides us with a useful analytic that apprehends a specific differentiating technology of predatory colonialism and capitalism, one that utilises identity checks, racialised surveillance, deportation and restrictive welfare policy, and that requires patriarchal subjugation, racial divisions and hierarchies, and class-based labour exploitation. As such, I would argue, *No Pass Laws Here!* also gestures towards important possibilities for international resistance and solidarity.

## Conclusion: The Global ‘Hostile Environment’

This article has sought to chart an earlier history of what we today call Britain’s hostile environment and a specific instance of resistance to it, offering new insights into the history of anti-racist border resistance in the UK. Taking the understudied *No Pass Laws Here!* Group’s materials as both an archive that demonstrated the earlier history of internal border controls and as an analytic of such controls, I have suggested that the group offer a situated anti-imperialist working-class analysis that allows us to understand internal border controls as a specific colonial technology of governance that facilitates capitalist extraction and that have endured over time. Here I have shown that activists based in the UK drew connections between racialised surveillance and governance in the metropole (UK) and colonies (South Africa). In so doing, I argue that the Group’s campaigning offers a situated analysis of internal border controls in 1980s UK while also urging us to construct a history of such borders that draws connections between different national contexts. Conceptually, this allows us to situate internal border controls as an instance of (post-)colonial ordering.

From 1988 onwards, the *No Pass Laws Here!* Group’s activities petered out and their bulletins ceased production. Yet their activities, namely documenting the spread and impacts of Britain’s border regime, continued through the work of their constituent members including the many law centres and migrant organisations such as the Joint Council for the Welfare of Immigrants, who continue to play a key role in documenting and resisting the hostile environment today (JCWI, n.d.). Nonetheless, the significance of this archive today, I want to suggest, should not be understated. The *No Pass Laws Here!* archive offers us a rich insight into the understudied history of internal border controls in the UK and resistance to them, a history that can be understood as a precursor to today’s hostile environment policies and that also places such policies in genealogical relation to colonial modes of governance.

As the UK’s hostile environment policies and the detention and deportation regime persist, having expanded to include off-shore detention sites, the archival materials and analysis presented in this article offer insights into previous bordering policies, which remain applicable today. In detailing an earlier history of internal bordering, this article points to how internal border controls create exploitable racialised social divisions that can function to divide workers and welfare recipients and to position migrant and/or racialised workers with differentiated lesser access to state resources and social life. Through connecting internal border controls in 1980s Britain to the pass law regime of apartheid South Africa, I have further suggested that the group allow for connections to be made between the local violence of internal borders in Britain and a broader global network of racialised differentiation, extraction and dispossession that work to sustain flows and networks of capital. In so doing, I suggested that *No Pass Laws Here!* as a demand helps us move from a nation-centric analysis to one that recognises the continuities of various struggles against state and border violence.

As such, I want to conclude by suggesting that *No Pass Laws Here!* as a demand operates as a refusal of the politics of fragmentation that characterises the neat nation-centric analysis of individual border regimes, instead invoking a politics of resistance at a local level that is intertwined with a politics care for those subjugated elsewhere. Rather than universalise the race–capitalism–nationalism nexus which gives internal borders their modern force, this analysis is intended to point to a particular set of tactics and technologies of governance and control that manifest in both post-colonial Britain and other (post-)colonies. In Palestine, for example, scholars have intricately documented how the wall that divides the West Bank from Jerusalem and Palestine 1948, along with its associated checkpoints and permit regime, functions to both dispossess, control and subjugate the Palestinian population while also ‘producing a docile male Palestinian labour force to build settlements for the Israeli population’ (Griffiths and Repo, 2018: 19). While the Kafala system of migrant visa sponsorship prevalent in the Gulf states, a ‘product of British colonial practices to control labour and police empire across the Gulf and the Indian Ocean’ (AlShehabi, 2019: 310), today operates as a racialised and gendered system of labour exploitation and subjugation (Fernandez, 2021; Pande, 2013). These are two examples of many, and the varied creation and control of exploitable and deportable labour reminds us that the hostile environment policies today are not new or singular, but rather that they are imbricated and connected to these histories and regimes and as such our resistance must hold them in common. Thus, in revisiting the demand and the call – *No Pass Laws Here!* – this article has sought to highlight these shared histories that demanded nothing short of the abolition, both here and elsewhere, of regimes of state racism, surveillance and border controls.

## Notes

1. Various campaigns have arisen to challenge the internal border policies associated with the hostile environment including, Patients not Passports, Docs Not Cops, Against Borders for Children and Unis Resist Border Controls. In addition, already existing migrant, anti-racist and community originations have developed specific campaigns and services that seek to resist internal border policies, including The Joint Council for the Welfare of Immigrant (JCWI), Migrants Organise, Liberty, Doctors of the World and North East London Migrant Action. Alongside these groups, Caribbean diplomats and politicians, including Guy Hewitt (Barbados), Keith Mitchell (Grenada) and Gaston Browne (Antigua and Barbuda), raised concerns with the UK government regarding the detention and deportation of members of the Windrush generation, which was facilitated by the hostile environment policies.

2. *No Pass Laws Here!* Bulletin No. 1, January 1982, P.2 (Institute of Race Relations (IRR) 01-04-03-02-122).

3. *No Pass Laws Here!* Bulletin, May 1987, P.2 (IRR 01-04-04-01-08-07-52).

4. *No Pass Laws Here!* Bulletin, May 1987, P.2 (IRR 01-04-04-01-08-07-52).

5. *No Pass Laws Here!* Bulletin No. 1, January 1982, P.2 (IRR 01-04-03-02-122).

6. ‘Racism in social security’, *No Pass Laws Here!* Bulletin No. 2, March 1982 (Hull History Centre (HHC) U DJC/23/3).

7. ‘Racism in social security’, *No Pass Laws Here!* Bulletin No. 2, March 1982 (HHC U DJC/23/3).

8. *No Pass Laws Here!* Leaflet, 1982 (HHC U DJC/23/1).

9. ‘Britain’s “pass laws”’, *No Pass Laws Here!* Bulletin, May 1987 (IRR 01-04-04-01-08-07-52).

10. ‘Internal immigration controls’, Migrants Action Group, April 1982 (HHC U DJC 23/1).

11. ‘Racism in social security’, *No Pass Laws Here!* Bulletin No. 2, March 1982 (HHC U DJC/23/3).

12. ‘Racism in social security’, *No Pass Laws Here!* Bulletin No. 2, March 1982 (HHC U DJC/23/3).

13. ‘Britain’s “pass laws”’, *No Pass Laws Here!* Bulletin, May 1987 (IRR 01-04-04-01-08-07-52).

14. ‘Charges for healthcare’, *No Pass Laws Here!* Bulletin No. 1, January 1982, P.1 (IRR 01-04-03-02-122).

15. ‘Charges for healthcare’, *No Pass Laws Here!* Bulletin No. 1, January 1982, P.1 (IRR 01-04-03-02-122).

16. ‘Internal controls at work’, *No Pass Laws Here!* Bulletin No. 4, October 1982 (HHC U DJC/23/1).

17. ‘Internal controls at work’, *No Pass Laws Here!* Bulletin No. 4, October 1982 (HHC U DJC/23/1).

18. ‘Internal controls at work’, *No Pass Laws Here!* Bulletin No. 4, October 1982 (HHC U DJC/23/1).

19. Pass laws as system or technology of colonial rule and racialised labour regulation were not unique to South Africa and were present within other colonial states on the African continent, including Zimbabwe. I focus on South Africa’s pass laws because the *No Pass Laws Here!* Group made direct reference to them in the public-facing materials.

20. *No Pass Laws Here!* Leaflet, 1982 (HHC U DJC/23/1).

21. ‘Britain’s “pass laws”’, *No Pass Laws Here!* Bulletin, May 1987 (IRR 01-04-04-01-08-07-52).

22. Azania Solidarity Campaign, January 1982 (HHC U DJC/23/1).

23. The language of pass laws can also be found in campaign materials produced by various anti-deportation and anti-police brutality campaigns. For example, in 1982 the Newham 8 Defence Campaign deployed the notion of pass laws in their critique of police brutality and British state racism.
